# Letter to the editor: Development and validation of a machine learning-based risk model for metastatic disease in nmCRPC patients: a tumor marker prognostic study

**DOI:** 10.1097/JS9.0000000000003410

**Published:** 2025-09-10

**Authors:** Yushan Jin, Yuan Fang, Yao Yao

**Affiliations:** a Department of Laboratory Medicine The First Affiliated Hospital of Dalian Medical University, Dalian, China; b College of Laboratory Medicine, Dalian Medical University, Dalian, China; c Department of Pharmacy, The First Affiliated Hospital of Dalian Medical University, Dalian, China

*Dear Editor,*

We read with great interest the article titled “Development and validation of a machine learning-based risk model for metastatic disease in nmCRPC patients: a tumor marker prognostic study” published in the *International Journal of Surgery*, authored by Ni *et al*[1]. We highly appreciate the innovative development and validation of a machine-learning-based metastasis risk prediction model for patients with nonmetastatic castration-resistant prostate cancer (nmCRPC). This work has far-reaching implications for the precise management of patients with nmCRPC in current clinical practice. We hereby declare that this manuscript was translated and linguistic checked using artificial intelligence to ensure compliance with journal requirements. The TITAN Guideline checklist was submitted according to the requirements of the literature “Transparency In the reporting of Artificial Intelligence-The TITAN guideline”[2].

The high risk of progression to metastasis and poor prognosis in patients with nmCRPC represents an important challenge in the field of urology[3]. The lack of validated prognostic models to accurately predict the probability of metastasis in these patients has long made it difficult for clinicians to stratify risk, develop follow-up strategies, and select personalized treatment intensity[4]. Given the significant impact of metastatic disease on overall survival (OS) and the validated importance of metastasis-free survival (MFS) as a surrogate endpoint for OS in patients with metastatic castration-resistant prostate cancer (mCRPC)[5], the study by Ni *et al* fills this gap. The results are expected to provide a powerful tool for clinical decision-making.

This study has key strengths in its robust data and advanced methods. Researchers integrated data from 2716 nmCRPC patients. These data came from two large, independent Phase III trials: SPARTAN (NCT01946204) and ARAMIS (NCT02200614)^[6-9]^. This approach established a large, representative cohort. Using multicenter clinical trial data significantly enhance the model’s generalizability. It also boosts the reliability of the model’s external validation. Furthermore, the research team employed multiple machine learning algorithms. They ultimately selected the LASSO and Stepwise Cox approach as the optimal solution. This rigorous method is widely adopted in prognostic model development. The model demonstrated excellent predictive performance. Its C-index was 0.724 in internal validation. In external validation, its time-dependent AUC remained ≥ 0.70 from 6 to 39 months. The model also showed strong calibration and clinical utility. Decision curve analysis confirmed these benefits. This comprehensive process ensures the model’s robustness and reliability.

This study identified eight significant prognostic variables, encompassing novel hormone agents, Gleason score, treatment history (prior surgery/radiation or none), Caucasian race, prostate specific antigen (PSA) doubling time, hemoglobin (HGB), and IgPSA[10]. Beyond conventional prognostic markers, it newly recognized the significance of advanced hormonal therapies (e.g., apalutamide, enzalutamide), prior intervention, race, and HGB concentration. These elements contribute to a broader understanding of disease progression in nmCRPC.

The model further introduces two clinical stratification approaches: one relying on computed risk scores, the other on the count of risk factors present. Using either method – classifying patients into low-, intermediate-, or high-risk categories based on score, or grouping by factor count – significant disparities in MFS and OS were consistently observed. Compared to the low-risk group, intermediate- and high-risk cohorts exhibited markedly elevated metastasis hazard ratios of 1.72 and 4.43, respectively, affirming the model’s discriminative capacity. Such refined risk classification supports personalized clinical decision-making, potentially enhancing outcomes while minimizing overtreatment. Additionally, the model offers a structural basis for future trial design by improving patient selection according to required treatment intensity[11].

Despite these advances, we also acknowledge the limitations of this discussion. For instance, the exclusion of patients from recent trials owing to missing values may introduce selection bias. Additionally, while the included variables were clinically justified and statistically significant in the univariate analyses, the model’s performance in real-world populations may differ from that of the clinical trial cohort. The authors appropriately noted the model’s primary derivation from patients with PSA doubling times <10 months, potentially limiting generalizability to lower-risk populations (e.g., PSA doubling time >10 months or Gleason score 3 + 3). The discussion also thoughtfully addresses the emerging role of novel molecular imaging techniques (e.g., Prostate-specific membrane antigen positron emission tomography, PSMA PET), concluding that current prognostic stratification in nmCRPC remains primarily guided by conventional imaging due to insufficient high-level evidence for the integration of PSMA PET into risk models. These considerations reflect the authors’ scientific rigor and highlight future research priorities: external validation in diverse real-world cohorts and exploration of novel biomarkers and advanced imaging data.

In conclusion, the machine learning-based metastatic risk prediction model for nmCRPC patients developed by Ni *et al* represents a significant and timely contribution. Its methodological robustness and high clinical utility potential are poised to enhance risk stratification and personalized management in nmCRPC. We anticipate that this model will positively influence both clinical practice and future research in this field.

## Data Availability

Data sharing is not applicable to this article as no new datasets were generated or analysed during the current study.

## Research registration unique identifying number (UIN)

Not applicable. This study does not involve human participants and therefore does not require registration.
